# Features of Maternal HIV-1 Associated with Lack of Vertical Transmission

**DOI:** 10.2174/1874357901710011008

**Published:** 2017-03-23

**Authors:** Nafees Ahmad, Aamir N. Ahmad, Shahid N. Ahmad

**Affiliations:** Department of Immunobiology, College of Medicine, The University of Arizona Health Sciences Center, Tucson, Arizona 85724, USA

**Keywords:** HIV-1, Vertical transmission, Maternal HV-1, HIV-1 structural genes, HIV-1 accessory genes

## Abstract

HIV-1 is transmitted from mother-to-child (vertical transmission) at an estimated rate of approximately 30% without any antiretroviral therapy (ART). However, administration of ART during pregnancy considerably diminishes the rate of mother-to-child transmission of HIV-1, which has become a standard of perinatal care in HIV-infected pregnant females in developed countries. Moreover, a majority of children born to HIV-infected mothers are uninfected without any ART. In addition, characteristics of HIV-1 and/or cellular factors in the mothers may play a role in influencing or preventing vertical transmission. Several studies, including from our laboratory have characterized the properties of HIV-1 from infected mothers that transmitted HIV-1 to their infants (transmitting mothers) and compared with those mothers that failed to transmit HIV-1 to their infants (non-transmitting mothers) in the absence of ART. One of the striking differences observed was that the non-transmitting mothers harbored a less heterogeneous HIV-1 population than transmitting mothers in the analyzed HIV-1 regions of p17 *gag*, *env* V3, *vif* and *vpr*. The other significant and distinctive findings were that the functional domains of HIV-1 *vif* and *vpr* proteins were less conserved in non-transmitting mothers compared with transmitting mothers. Furthermore, there were differences seen in two important motifs of HIV-1 Gag p17, including conservation of QVSQNY motif and variation in KIEEEQN motif in non-transmitting mothers compared with transmitting mothers. Several of these distinguishing properties of HIV-1 in non-transmitting mothers provide insights in developing strategies for preventing HIV-1 vertical transmission.

## INTRODUCTION

The rate of mother-to-child transmission (vertical) of HIV-1 is about 30% when infected mothers are not treated with antiretroviral drugs during pregnancy [1-5]. Vertical transmission primarily takes place at three steps: prepartum through transplacental passage, intrapartum where the infants skin and mucus membrane are exposed to mother’s blood and vaginal secretions, and postpartum through breast milk. HIV-1 vertical transmission is influenced by several factors in the mothers such as high viral load, low CD4 T cell counts, symptomatic HIV disease, immune response, recent infections and heterogeneous HIV-1 population [1-5]. However, antiretroviral treatment (ART) during pregnancy has significantly reduced HIV-1 vertical transmission rates [6] mainly in the developed countries. More importantly, HIV-1 mother-to-child transmission is still a grave concern in many developing countries where much of HIV infection in children occurs through vertical transmission [7, 8].

## CHARACTERIZATION OF VIRAL DETERMINANTS ASSOCIATED WITH VERTICAL TRANSMISSION

Elucidation of viral determinants and maternal factors involved in HIV-1 vertical transmission has several merits for preventive strategies because more than two-third of the infants born to HIV-1 infected mothers are uninfected without any antiretroviral treatment. Several studies have shown that HIV-1 variants present in mothers that are transmitted to infants were not efficiently neutralized by maternal antibody [9, 10], including those maternal viral variants that are near the time of transmission [10]. We and others have characterized the molecular and biological properties of HIV-1 from mothers and their infants after vertical transmission, and demonstrated that a minor maternal HIV-1 variant was transmitted to their infants [11-16]. Several studies have shown a similar pattern of transmission of HIV-1 from transmitter to recipient during sexual transmission [17-19]. Furthermore, the biological phenotype of HIV-1 involved in both vertical and sexual transmission was shown to be consistently R5 HIV-1 [20-23].

In a more comprehensive approach to understand the molecular properties of HIV-1 that are associated with vertical transmission, we showed that there was a high degree of preservation of the functional domains of HIV-1 in the structural genes, including *gag* p17 [24] and NC [25], *pol* RT [26], *env* gp120 [13] and gp41 [27], regulatory genes *tat* [28] and *rev* [29] and their cis-acting responsive regions, HIV-1 LTR [30, 31] and RRE [32] and accessory genes, *vif* [33], *vpr* [34], *vpu* [35] and *nef* [36] from mother-infant pairs after vertical transmission. While most of these studies focused on elucidating the properties of HIV-1 associated with vertical transmission, limited studies have characterized the properties of HIV-1 that are associated with lack of vertical transmission. Better characterization of viral determinants and host factors associated with lack of vertical transmission could be critical in developing preventive strategies. Our group has performed a comparative analysis of the properties of HIV-1, including genetic variability and functional domains of HIV-1, between non-transmitting mothers (mothers who failed to transmit the virus to their infants in the absence of any antiretroviral therapy) and transmitting mothers (mothers who transmitted the virus to their infants). In this article, we will focus to compile the unique features of HIV-1 that are associated with lack of vertical transmission, especially in two important regions of the HIV-1 genome; the structural regions *gag* p17 and *env* V3 and accessory genes, *vif* and *vpr*.

## CHARACTERIZATION OF HIV-1 *gag* p17 ASSOCIATED WITH LACK OF VERTICAL TRANSMISSION

HIV-1 structural gene *gag* p17 matrix plays an important role in HIV-1 replication, viral determinants or motifs in *gag* p17 may be associated with and/or lack of vertical transmission. Therefore, our previous study analyzed the HIV-1 *gag* p17 sequences from non-transmitting mothers, including a mother with several births [37] and compared with our and others published study on transmitting mothers’ sequences [24, 38]. Our analysis revealed that there were intact Gag p17 matrix open reading frames in most of our non-transmitting [37] and transmitting mothers’ sequences [24]. We also compared the viral heterogeneity and found that the *gag* p17 matrix sequences in non-transmitting mothers [37] were less heterogeneous than transmitting mothers’ sequences [24]. With respect to variability in the nucleotide sequences of p17 matrix, the distance ranged from 0 to 5.6% (0% median values) and amino acid from 0 to 7.7% (0% median values) in non-transmitting mothers [37] compared transmitting mothers [24] where the nucleotide distances ranged from 0.2 to 6.5% (2.3% median values) and amino acid from 0 to 12.0% (4.5% median values). These findings supported the notion that a low degree of viral heterogeneity in infected mothers is associated with lack of vertical transmission.

Several motifs in Gag p17 matrix sequences, including glutamic acid (E) at position 93, KIEEEQN motif (positions 103-109, the major antibody binding site) were conserved more in transmitting than non-transmitting mothers [24, 37, 38]. In addition, a valine (V) at position 104 was shown to be present in non-transmitting status compared with transmitting mothers’ sequences [24, 38]. Several studies found that the C-terminal 6-mer QVSQNY, a lysine (K) or glutamine (Q) at position 15, an alanine (A) at 54, a lysine (K) at 76, a valine (V) at 104 and an aspartic acid (D) at 102 and 121 were associated with non-transmitting status [37, 38]. Our study also compared the motifs required for functional activity of Gag p17 matrix protein between non-transmitting and transmitting mothers’ sequences and found that the functions such as targeting of Gag to the plasma membrane, virus assembly and release, envelope glycoprotein incorporation into the virion, and localization of the virus preintegration complex to the nucleus of nondividing cells [39-42] were largely preserved in non-transmitting [37] and transmitting mothers’ [24] sequences. Another functional domains of Gag p17 matrix namely the polymerization site located at positions 47 to 59 was less conserved in non-transmitting mothers’ sequences [37] than transmitting mothers’ sequences [24]. Some of these motifs in *gag* p17 matrix that differ between transmitting and non-transmitting mothers could be targeted for preventive strategies of HIV vertical transmission.

## CHARACTERIZATION OF HIV-1 *env* V3 REGION ASSOCIATED WITH LACK OF VERTICAL TRANSMISSION

Comparison of the V3 region of HIV-1 envelope gene, which plays an important role in virus neutralization, cellular tropism, replication and pathogenesis [43-45], between non-transmitting and transmitting mothers’ sequences may provide important viral information related to vertical transmission. While the coding potential of the V3 region were highly conserved in non-transmitting [46] and transmitting [13-16] mothers’ sequences, the V3 region sequences of non-transmitting mother’s sequence [46] were significantly less heterogeneous compared with transmitting mothers’ V3 region sequences [13]. Furthermore, the estimates of genetic diversity of non-transmitting mothers’ V3 region sequences were significantly lower compared with transmitting mothers’ V3 region sequences [46]. The lower level of viral heterogeneity of V3 region sequences in non-transmitting mothers compared with transmitting mothers’ sequences correlated with lack of vertical transmission, as also seen in p17 *gag* sequences. Another striking feature in support of the correlation of less heterogeneity of HIV with lack of vertical transmission was the decreasing viral heterogeneity and rate of genetic diversity with successive births in non-transmitting mothers [46].

There were several amino acid sequence motifs present in non-transmitting mothers that were specific to each non-transmitting mother when compared to transmitting mothers [46] did not provide any specific pattern related to either associated with or lack of vertical transmission. However, examining the non-transmitting mothers and one non-transmitting mother with multiple deliveries suggested clearly that if HIV stays less heterogeneous, the mother-to-child transmission could be significantly reduced. This conclusion is further supported by the fact that the one non-transmitting mother’s HIV sequences were almost homogeneous at the time of her fifth delivery [46].

Mother-to-child transmission has been shown to be significantly reduced by use of ART, which suggests that suppression of HIV replication is achieved, that also reduces HIV heterogeneity because of suppression of HIV replication. A low level of HIV-1 heterogeneity associated with lack of mother-to-child of transmission [46] provides a logical explanation on the mechanism of reduction in HIV vertical by using ART during pregnancy. The use of ART is known to suppress HIV-1 replication and lowers viral load in infected patients [6, 47, 48], which also reduces viral heterogeneity because of no active viral replication in the presence of ART [48]. This decrease in HIV-1 heterogeneity due to ART in infected mothers most likely reduced HIV-1 mother-infant transmission. The lower estimates of genetic diversity in non-transmitting mothers’ HIV-1 populations joined with different levels of selection pressure [46] suggest the mothers’ ability to mount a different degree of effective immune response against the viral population and prevent transmission by neutralizing the virus. We saw in our study that HIV-1 sequences in the non-transmitting mothers were homogeneous [46], which provides an indication that the HIV-1 population has attained a state of optimum adaptation as seen in long-term progressors of HIV-1 infection [49] may also apply in the case of non-transmitting mothers. These findings of a more slowly evolving virus population in the V3 region provide a better target for developing strategies for mounting an immune response to control the infection and prevent transmission.

## CHARACTERIZATION OF HIV-1 *vif* AND *vpr* GENES ASSOCIATED WITH LACK OF VERTICAL TRANSMISSION

HIV-1 replication is regulated by several regulatory and accessory genes, including *vif* and *vpr* that are required for HIV-1 pathogenesis [50-52] and may also have some role in mother-to-child transmission. Therefore, a comparative analysis of HIV-1 *vif* and *vpr* genes from transmitting and non-transmitting mothers should provide useful information that may allow us to correlate the properties of these genes with HIV-1 vertical transmission, as shown in our studies [33, 34, 53]. In these studies, we have shown that the molecular motifs that are essential for the biological function of *vif* and *vpr* in HIV-1 were conserved in HIV-1 isolates from mothers and infants after vertical transmission [33, 34], suggesting that *vif* and *vpr* proteins are required for transmission and pathogenesis. In contrast, the functional domain of *vif* and *vpr* in non-transmitting mother isolates were less conserved [53], providing a first line of evidence that *vif* and *vpr* may have a role in HIV-1 vertical transmission. Another important finding was that *vif* and **vpr** sequences were less heterogeneous in non-transmitting HIV-1 isolates [53] than transmitting mothers [33, 34], which is consistent with a low level of heterogeneity seen in *gag* p17 [37] and *env* V3 region [46].

Some of the in-depth analysis of the deduced amino acid sequences of *vif* nucleotide sequences from non-transmitting HIV-1 isolates showed that some non-transmitting mothers *vif* sequences contained stop codons and lacked initiation codons [53], indicating that *vif* was not functional and therefore may have prevented mother-to-child transmission. One of the other important findings was that the *vif* sequences from some non-transmitting mothers [53] carried a substitution of histidine in place of tyrosine at position 30 in a highly conserved motif SL (I/V) X4YX9Y among HIV-1 isolates located between amino acid 23-40 that was preserved in our transmitting mothers [33]. Some other non-transmitting mothers [53] contained substitutions such as asparagine at position 22, lysine at 77 and histidine at 110 [50]. The substitutions of polar charged lysine to polar uncharged asparagine at position 22 and non-polar tyrosine to polar histidine at 30 and 110 are major changes and may affect *vif* function. The *vif* gene is highly conserved in all HIV-1 isolates in HIV-1 infected patients [50] as well as transmitting mother-infant pairs [33]. In contrast, the non-transmitting mothers’ *vif* sequences [53] were less functional, suggesting a role in preventing HIV-1 vertical transmission.

Upon examination of the deduced amino acid sequences of *vpr* in non-transmitting mothers’ HIV-1 isolates [53], we found that some non-transmitting mother *vpr* sequences had stop codons and lacked initiation codons, whereas some non-transmitting mothers’ *vpr* sequences contained a substitution of serine in place of alanine at position 30, arginine in place of glycine at position 75, and a deletion in the C-terminus necessary for *vpr* function. Some non-transmitting mother sequences had a substitution of arginine in place of glycine at position 75 of the conserved dipeptide (GC) that is important in virion incorporation and stability of *vpr* [54] and cell cycle arrest [55] and a serine in place of alanine at position 30 that is required for cell cycle arrests by *vpr* [55, 56]. More importantly, the *vpr* sequences from a non-transmitting mother from three time points contained an in-frame deletion at the C-terminus [53] that has been shown to affect cell cycle arrest property of *vpr* [55], which were also seen in long-term survivors of HIV-1 infection [57]. In summary, the functional domains of *vpr* in non-transmitting mother were either defective, less functional or contained substitution in important domains that affected *vpr* function compared with transmitting mothers *vpr* sequences that had all functional domains intact [53]. These data suggested that *vif* and *vpr* are important viral determinants of HIV-1 vertical transmission and defect in these genes may be associated with lack of vertical transmission, as found in non-transmitting mothers *vif* and *vpr* sequences [53].

## CONCLUSION

While HIV-1 mother-to-child (vertical) transmission of HIV-1 is multifactorial in nature, viral determinants may play an important role in either influencing or preventing vertical transmission. Several studies, including our study characterized the molecular and biological properties of HIV-1 associated with vertical transmission and showed that HIV-1 structural, regulatory and accessory genes were highly conserved and functional. We have performed several studies examining HIV-1 structural and accessory genes from infected mothers who failed to transmit the virus to their infants in the absence of any antiretroviral therapy. In this article, we have compiled the findings of our studies that showed that HIV-1 p17 *gag* matrix, envelope V3 regions, *vif* and *vpr* sequences were significantly less heterogeneous in non-transmitting mothers than transmitting mothers. In addition, the *vif* and *vpr* from non-transmitting mothers were either defective, less functional or had substitution in important domains affecting function, whereas transmitting mothers *vif* and *vpr* were intact and functional. These findings are consistent with the data showing that more than two-third of HIV-infected pregnant women do not transmit HIV to their infants more likely of their HIV-1 sequences being less heterogeneous and *vif* and *vpr* less functional. In addition, our findings are also consistent with the use of antiretroviral therapy in preventing HIV-1 vertical transmission because antiretroviral therapy suppresses viral replication and reduces viral heterogeneity. Our non-transmitting mothers harbored a homogenous HIV sequences, whereas transmitting mothers had a heterogeneous HIV sequences, suggesting that less HIV heterogeneity was associated with the lack of vertical transmission. These results provide an explanation for the reduction of vertical transmission by antiretroviral therapy (ART) during pregnancy, as ART is known to suppress virus replication and reduce viral load viral heterogeneity. These findings may have ramifications in combating vertical transmission by developing new therapies.
